# Lack of GPR180 ameliorates hepatic lipid depot via downregulation of mTORC1 signaling

**DOI:** 10.1038/s41598-023-29135-5

**Published:** 2023-02-01

**Authors:** Ken Yoshida, Kazuha Yokota, Kazuhisa Watanabe, Hidetoshi Tsuda, Ayumi Matsumoto, Hiroaki Mizukami, Sadahiko Iwamoto

**Affiliations:** 1 Division of Human Genetics, Center for Molecular Medicine, Jichi Medical University, 3311-1 Yakushiji, Shimotsuke, Tochigi, 329-0498 Japan; 2 Division of Genetic Therapeutics, Center for Molecular Medicine, Jichi Medical School, Tochigi, Japan

**Keywords:** Non-alcoholic fatty liver disease, Dyslipidaemias

## Abstract

Our previous genome-wide association study to explore genetic loci associated with lean nonalcoholic fatty liver disease (NAFLD) in Japan suggested four candidate loci, which were mapped to chr6, chr7, chr12 and chr13. The present study aimed to identify the locus involved functionally in NAFLD around the association signal observed in chr13. Chromosome conformation capture assay and a database survey suggested the intermolecular interaction among DNA fragments in association signals with the adjacent four coding gene promoters. The four genes were further screened by knockdown (KD) in mice using shRNA delivered by an adeno-associated virus vector (AAV8), and KD of *G protein-coupled receptor 180 (Gpr180)* showed amelioration of hepatic lipid storage. *Gpr180* knockout (KO) mice also showed ameliorated hepatic and plasma lipid levels without influencing glucose metabolism after high-fat diet intake. Transcriptome analyses showed downregulation of mTORC1 signaling and cholesterol homeostasis, which was confirmed by weakened phosphorylation of mTOR and decreased activated SREBP1 in *Gpr180*KO mice and a human hepatoma cell line (Huh7). AAV8-mediated hepatic rescue of GPR180 expression in KO mice showed recovery of plasma and hepatic lipid levels. In conclusion, ablation of GPR180 ameliorated plasma and hepatic lipid levels, which was mediated by downregulation of mTORC1 signaling.

## Introduction

Nonalcoholic fatty liver disease (NAFLD) is the most prevalent chronic hepatic disease in industrialized countries, and is defined as liver steatosis without alcoholic liver injury or other liver disease from apparent pathogenic factors^1^. The pathogenesis of NAFLD has not been fully resolved, but a complex interaction among environmental factors (i.e., western diet, obesity or intestinal microbiota) and genetic predisposition is thought to be involved in disturbed lipid homeostasis and excessive lipid accumulation in hepatocytes^2^. The heritability of NAFLD has been estimated to range from 20 to 70% in population-based and familial-aggregation studies^3^. Many studies have been conducted to explore the genetic factors associated with NAFLD, and several loci have been identified as deeply associated genetic factors through genome-wide association studies (GWAS)^4,5^. Some of the associated loci have been validated functionally. For example, a common missense variant (I148M) of *patatin-like phospholipase domain containing 3 (PNPLA3)* disrupts lipolytic activity on lipid droplets in hepatocytes and increases hepatic lipid deposition^6^. Further discovery of molecular pathways may support development of effective tool for the management of NAFLD.

Our previous GWAS to explore genetic loci associated with lean NAFLD in the Japanese population suggested four candidate loci, which were mapped to *HLA* in chr6, *MIR548F3* in chr7, *MYL2* in chr12 and *GPC6* in chr13^7^. The HLA locus was further analyzed and the involvement of the risk allele in enteric dysbiosis was shown as a NAFLD disposition mechanism. However, the other three loci remain to be analyzed. The present study aimed to identify the locus involved functionally in NAFLD around the association signal observed in chr13, which was mapped in intron6 of *GPC6*. In the adjacent loci, *GPR180* was emerged as a novel candidate gene involved in hepatic lipid deposition.

## Results

### Intron 6 of GPC6 interacts with adjacent gene promoter elements

**Figure 1:**
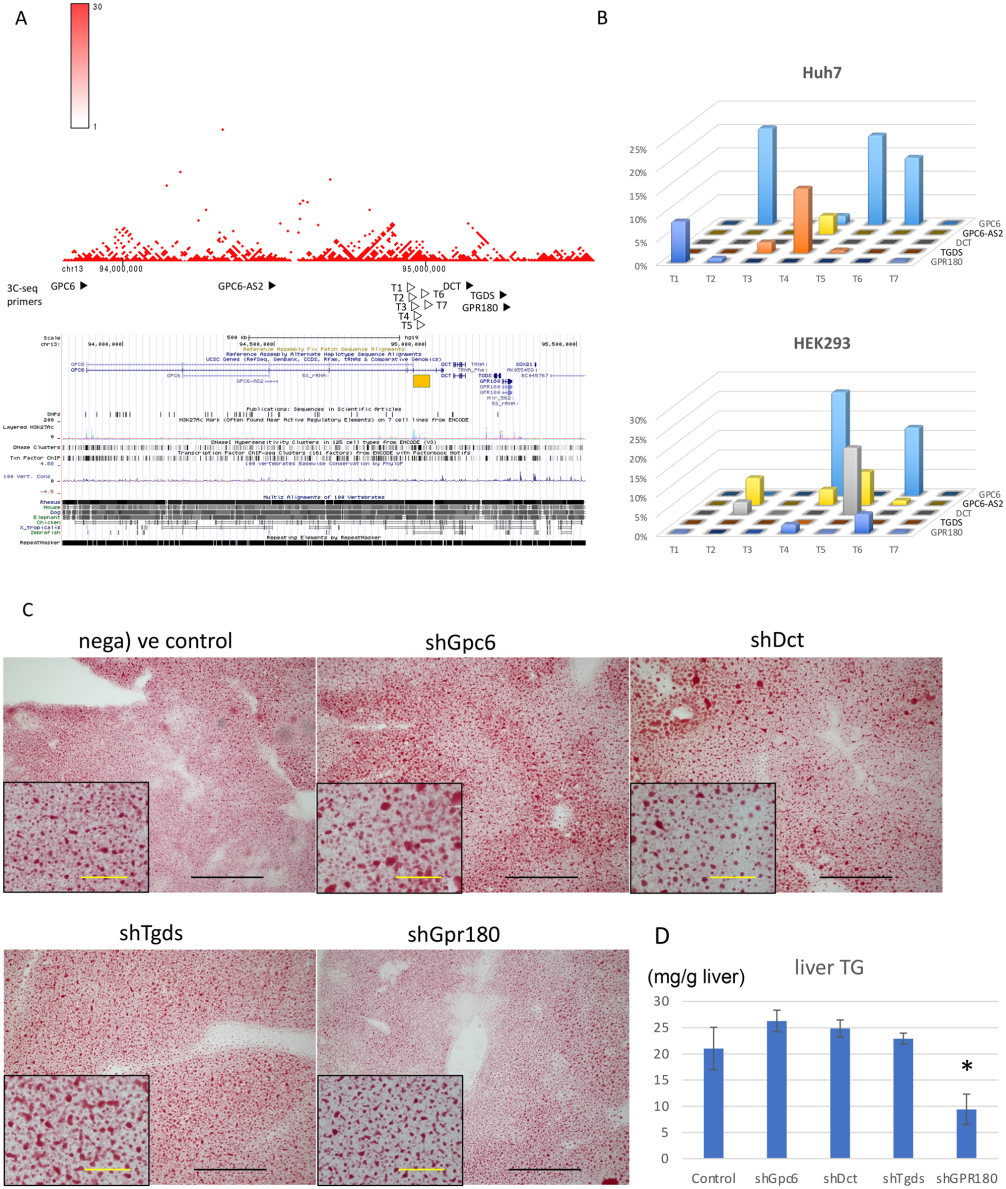
Bioinformatics and functional assessment of genetic association signals. (**A**) Bioinformatics data of HiC and ENCODE databases were merged on the identical chromosomal position around the GWAS association peak on chr13 (yellow bar). Open and closed triangles indicate the primer position used in the chromosome conformation capture assay (3C-assay). (**B**) Relative quantification of the 3C-assay. Crosslinked nuclei of Huh7 and HEK293 cells were digested with *Mbo*I and intermolecularly ligated. The ligation products were quantified using low cycle-PCR and subsequent pair-end MiSeq sequencing. Three-dimensional bar graphs show the relative amounts of PCR products amplified by the primer sets indicated in the X and Y axes. (**C**) Knockdown screening of the four coding genes. AAV8 vectors encoding shRNA templates for *Gpc6, Dct, Tgds, Gpr180* or negative control vector were injected into the tail vein of C57B6 mice. The vector-injected mice were fed with MCDD for one week. The extracted liver sections were stained by Oil Red O. Black and yellow scale bars indicate 100 and 20 μm, respectively. (**D**) Triglyceride (TG, mg/g liver) contents in the liver of each vector-injected group. Data are presented as means with standard deviation (n = 4 per AAV vector). *p < 0.05.

The ENCODE database shows a histone H3K27 acetylation mark in intron 6 of *GPC6*, which is a genome position identical to the association signal we previously observed^7^. Furthermore, DNaseI hypersensitivity marks and chromatin immunoprecipitation signals with several transcription factors were mapped in the same region, suggesting that this region regulates the adjacent genes. To identify the target gene of the intron 6 susceptible element, the chromatin interaction database of Hi-C was searched in human liver STL011 and lymphocyte GM12878 (http://3dgenome.fsm.northwestern.edu). In both tissues, intron 6 of *GPC6* interacts not only with the *GPC6* promoter, but also with the downstream genes including *DCT*, *TGDS* and *GPR180* (Fig. 1A). We then conducted a quantitative assay of chromosome conformation capture assay using Huh7 and HEK293 cells. Reads per sample in triplicate assays were 170,300 ± 7685 in Huh7 cells and 163,775 ± 5006 in HEK293 cells. While the candidate elements in intron 6 of *GPC6* interact mainly (Huh7 cells 55.8% and HEK293 cells 43.9%) with the *GPC6* promoter, interaction with *GPR180* was also observed (Fig. 1B). Simultaneously, the cell type specific manner of the interaction between elements in intron 6 and promoters suggested that the DNA region functions as a locus control region for the adjacent genes of *GPC6, GPC6-AS2, DCT, TGDS* or *GPR180*.

### Knockdown of Gpr180 expression attenuated hepatic lipid levels

To explore the genes associated with hepatic lipid levels in the protein coding genes interacting with *GPC6* intron 6, shRNA templates for *Gpc6, Dct, Tgds or Gpr180* were delivered to the mouse liver by *i.v.* injection of the AAV8 vector. After one-week feeding of methionine and choline-deficient diet (MCDD), mice were sacrificed and hepatic fat accumulation was evaluated using Oil Red O staining (Fig. 1C) and triglyceride (TG) measurement using Folch’s method (Fig. 1D). Both methods suggested significant attenuation of hepatic lipid accumulation in *Gpr180* KD mice compared to mice injected with negative control vector. The target specific decrease of the transcripts by shRNA were validated by qPCR (Supplementary Fig. 1).

### Global Gpr180 knockout mice attenuated hepatic lipid accumulation

**Figure 2:**
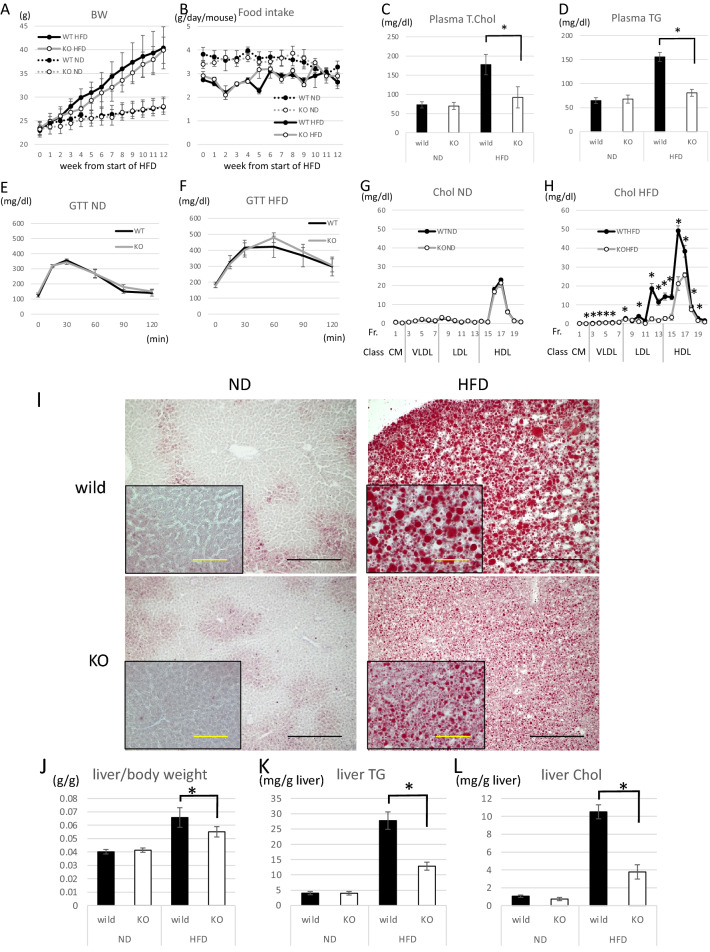
Phenotype characterization of *Gpr180*KO mice. (**A**) Body weight and (**B**) Food intake of wild-type and *Gpr180*KO (KO) mice fed high-fat diet (HFD) or normal diet (ND) for 12 weeks. Data are presented as means with standard deviation (n = 7). (**C**) Plasma cholesterol and (**D**) triglyceride (TG) levels in mice fed the indicated diets for 12 weeks and starved 4 h prior to sample harvesting. Data are presented as means with standard deviation (n = 7). (**E**) Intraperitoneal glucose tolerance test (IP-GTT) in 20-week-old male mice fed ND (n = 6). (**F**) IP-GTT in 20-week-old mice fed HFD (n = 6). (**G**) Cholesterol levels in HPLC-fractionated plasma of wild-type and KO mice fed ND (n = 4) or (**H**) HFD (n = 4). (**I**) Representative Oil Red O staining of 20-week-old mouse liver fed ND or HFD. Black and yellow scale bars indicate 100 and 20 μm, respectively. (**J**) Liver weight per body weight of wild-type and KO mice fed ND or HFD (n = 8). (**K**) TG and (**L**) cholesterol contents in liver extract (mg/g) of wild-type and KO mice fed ND or HFD (n = 8). *p < 0.05.

To further evaluate the effect of GPR180 deficit, whole body *Gpr180* knockout (KO) mice were generated. Thirty-four mice of forty-eight founder mice showed a deletion allele by tail DNA templated PCR (Supplementary Fig. 2C). Homozygous *Gpr180*KO mice (*Gpr180*KO) and control wild-type mice were established by mating of heterozygous founder mice after at least four backcrosses. There were no differences in gross phenotype between *Gpr180*KO and wild-type mice. Under normal diet (ND) feeding, body weight, plasma cholesterol, TG, and glucose levels were equivalent to those of wild-type mice (Fig. 2A–E,G); however, after 12 weeks of high-fat diet (HFD) feeding, plasma cholesterol level and TG were significantly lower than in wild-type mice (Fig. 2C,D). Cholesterol levels of *Gpr180*KO mice were reduced in all cholesterol fractions (Fig. 2H). Although body weight, food intake and glucose tolerance were equivalent between the two mouse groups even after HFD feeding (Fig. 2A,B,F), hepatic lipid depots (Fig. 2I), liver weight (Fig. 2J), hepatic TG and cholesterol (Fig. 2K,L) of *Gpr180*KO mice were significantly lower than in wild-type mice after 12 weeks of HFD feeding.

### Pathway analysis

**Figure 3:**
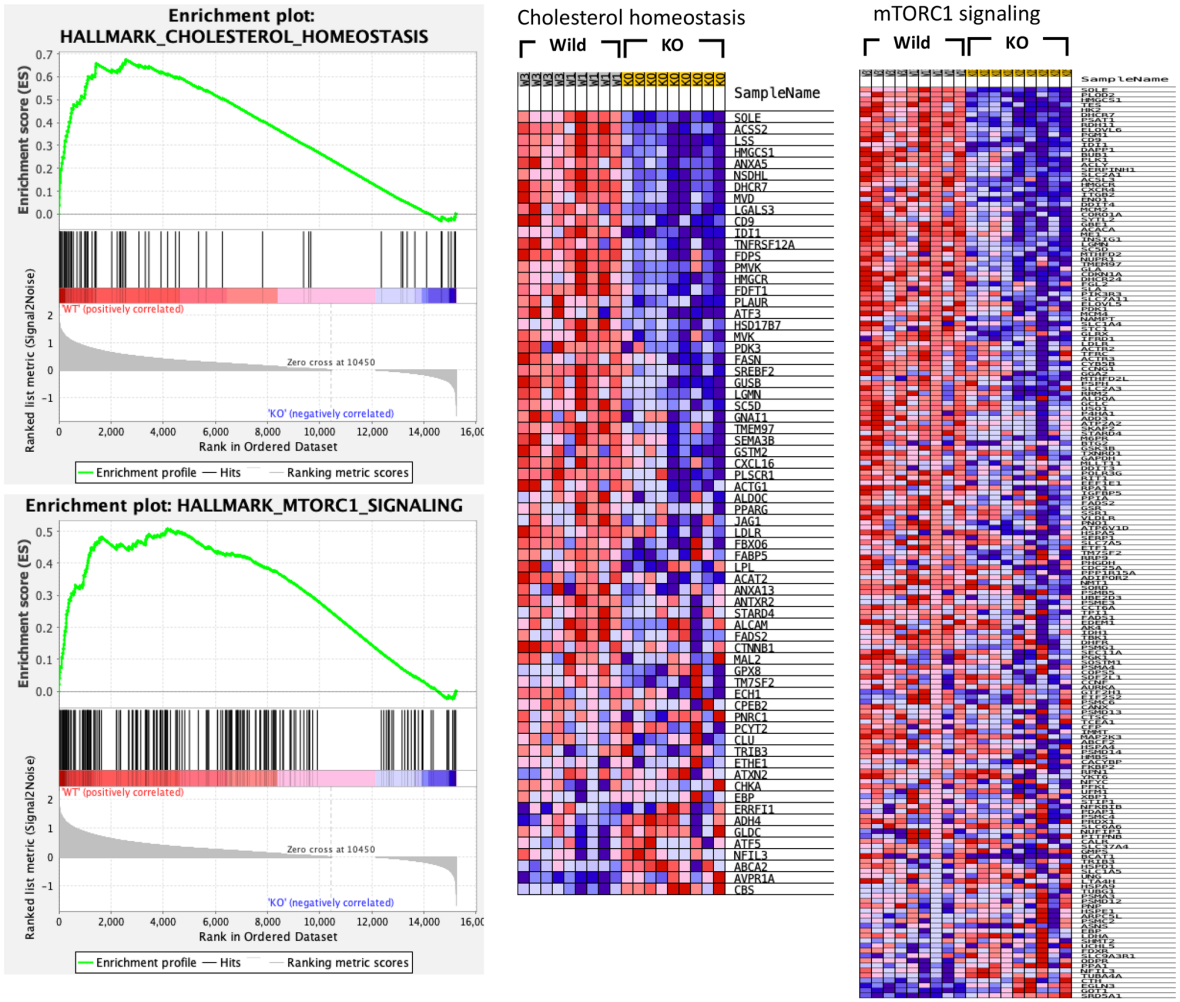
Gene set enrichment analysis of liver RNA sequences in the liver of wild-type and KO mice. Enrichment plots and heat maps are shown for gene sets of cholesterol homeostasis and mTORC1 signaling.

To explore the mechanism of attenuated lipid levels in *Gpr180*KO mice, whole transcriptome of wild-type and *Gpr180*KO mouse livers under ND and HFD feeding were analyzed with the GSEA program using the Hallmark database. The expression level of truncated *Gpr180* mRNA in *Gpr180*KO mice liver was reduced to 17% of the wild-type in fragments per kilobase of exon per million reads mapped (FPKM) level, while qPCR-based measurement showed further reduced (less than one-third) mRNA level in multiple organs in *Gpr180*KO mice (Supplementary Fig. 2D,E). *Gpr180*KO mice showed significantly reduced expression of the gene sets in cholesterol homeostasis and several signaling pathways (Fig. 3, Table 1), in which the mTORC1 signaling pathway is known to be involved in lipid metabolism. Down-regulation of gene expression involved in cholesterol biosynthesis was validated by real-time PCR. In addition, expression of TG biosynthesis and lipid transport genes was simultaneously reduced in *Gpr180*KO mice under HFD (Supplementary Fig. 3). Of the lipogenic transcription factors, *Srebf1* and *Srebf2* were down-regulated in *Gpr180*KO mice in accordance with the reduced expression of lipogenic genes.

**Table 1:** Gene sets enriched in wild type mice.

NAME	NES	FDR q-val
HALLMARK_CHOLESTEROL_HOMEOSTASIS	1.877	0.013
HALLMARK_EPITHELIAL_MESENCHYMAL_TRANSITION	1.787	0.019
HALLMARK_ANGIOGENESIS	1.781	0.015
HALLMARK_COAGULATION	1.775	0.013
HALLMARK_KRAS_SIGNALING_UP	1.753	0.013
HALLMARK_TNFA_SIGNALING_VIA_NFKB	1.745	0.013
HALLMARK_APOPTOSIS	1.735	0.011
HALLMARK_ESTROGEN_RESPONSE_LATE	1.706	0.012
HALLMARK_ESTROGEN_RESPONSE_EARLY	1.680	0.015
HALLMARK_IL6_JAK_STAT3_SIGNALING	1.678	0.014
HALLMARK_HYPOXIA	1.665	0.014
HALLMARK_APICAL_SURFACE	1.661	0.013
HALLMARK_ALLOGRAFT_REJECTION	1.655	0.012
HALLMARK_GLYCOLYSIS	1.650	0.012
HALLMARK_MTORC1_SIGNALING	1.638	0.013
HALLMARK_APICAL_JUNCTION	1.638	0.012
HALLMARK_COMPLEMENT	1.630	0.012
HALLMARK_MYOGENESIS	1.628	0.012
HALLMARK_INFLAMMATORY_RESPONSE	1.628	0.011
HALLMARK_IL2_STAT5_SIGNALING	1.622	0.011
HALLMARK_G2M_CHECKPOINT	1.543	0.026
HALLMARK_E2F_TARGETS	1.539	0.026
HALLMARK_NOTCH_SIGNALING	1.527	0.028
HALLMARK_UV_RESPONSE_UP	1.503	0.035
HALLMARK_P53_PATHWAY	1.494	0.037
HALLMARK_UV_RESPONSE_DN	1.460	0.050

Data with false discovery rate (FDR) q < 0.05 are shown. NES; normalized enrich score.

**Figure 4:**
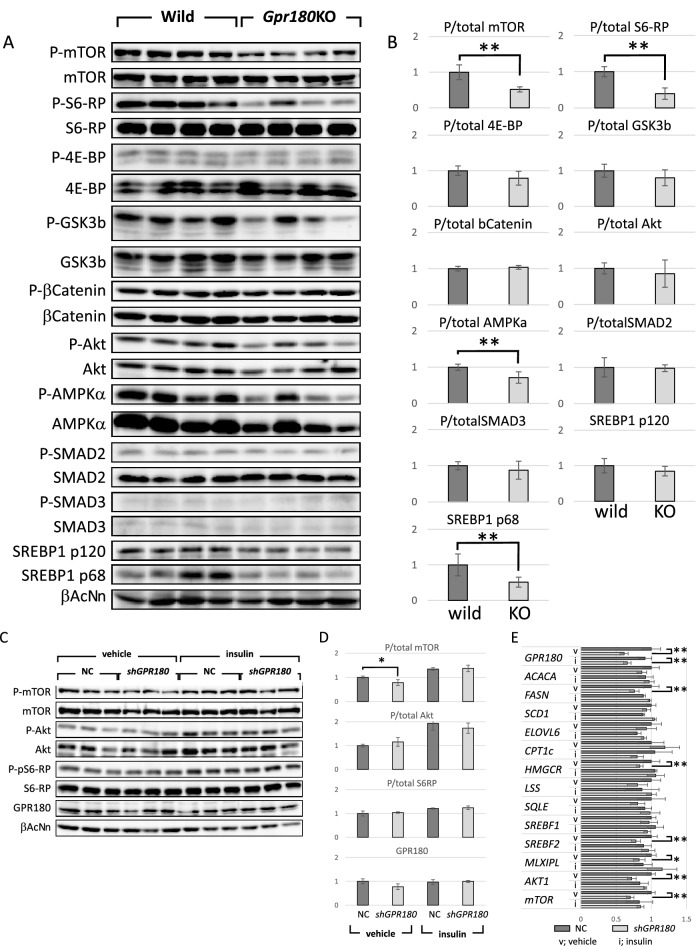
Western blotting and relative quantification of mouse liver proteins, and validation using a human hepatoma cell line. (**A**) Twenty micrograms of protein per liver sample of wild-type and KO mice were stained with the primary antibodies indicated on the left and HRP-labeled secondary antibody. (**B**) Relative band intensities against the normalized mean value of wild-type mice are shown as mean values with standard deviation (n = 8). (**C**) Twenty micrograms per sample of Huh7 cells transfected with pAAV-U6-negative control (NC) or pAAV-U6-shGPR180 plasmid for 24 h and subsequently stimulated with 100 nM insulin or without insulin (vehicle) were stained with the primary antibodies indicated on the left. (**D**) Relative band intensities against the normalized mean value of NC are shown as mean values with standard deviation (n = 6). (**E**) Relative mRNA levels in Huh7 cells against the normalized mean value of NC are shown as mean values with standard deviation (n = 6). *p < 0.05. **p < 0.01.

To clarify the actual signal modification by *Gpr180*KO, phosphorylation of signaling proteins nominated by GSEA was analyzed in the livers of HFD mice. Phosphorylation of mTOR was reduced in *Gpr180*KO mice (Fig. 4A,B). Although, reduction of phospho-4E-BP was subtle, p-S6 ribosomal protein (S6-RP) was significantly attenuated by the lack of GPR180, supporting the negative regulation of mTORC1 signaling. Furthermore, the activated forms of SREBP1 were simultaneously decreased in *Gpr180*KO mice. Phosphorylation of AMPKα was also reduced in *Gpr180*KO mice, but it was estimated that deactivation of AMPKα was resulted from weakened negative feedback loop against lower hepatic lipid accumulation than wild mice. However, phosphorylation of GSK3β, β-catenin, Akt, SMAD2 and 3 was not affected by ablation of *Gpr180*.

Reduction of mTOR phosphorylation was also shown in Huh7 cells, a human hepatoma cell line (Fig. 4C,D), which were knocked down by *GPR180* shRNA. While GPR180 mRNA levels were reduced by shRNA toward 61 to 66% of control vector, reduction of the protein levels was not significant. Reduction of phosphorylation of S6-RP was not shown either, probably due to the less effective shRNA. Insulin enhanced the phosphorylation of mTOR, Akt and S6-RP, but the difference of mTOR phosphorylation by *GPR180*KD was obscured. RNA levels of lipogenic genes, *AKT1* and *mTOR* were reduced in *GPR180* KD cells without insulin stimulation (Fig. 4E).

### AAV-mediated hepatic expression of GPR180 in knockout mice partially recovered plasma cholesterol and hepatic lipid accumulation, and mTOR phosphorylation

**Figure 5:**
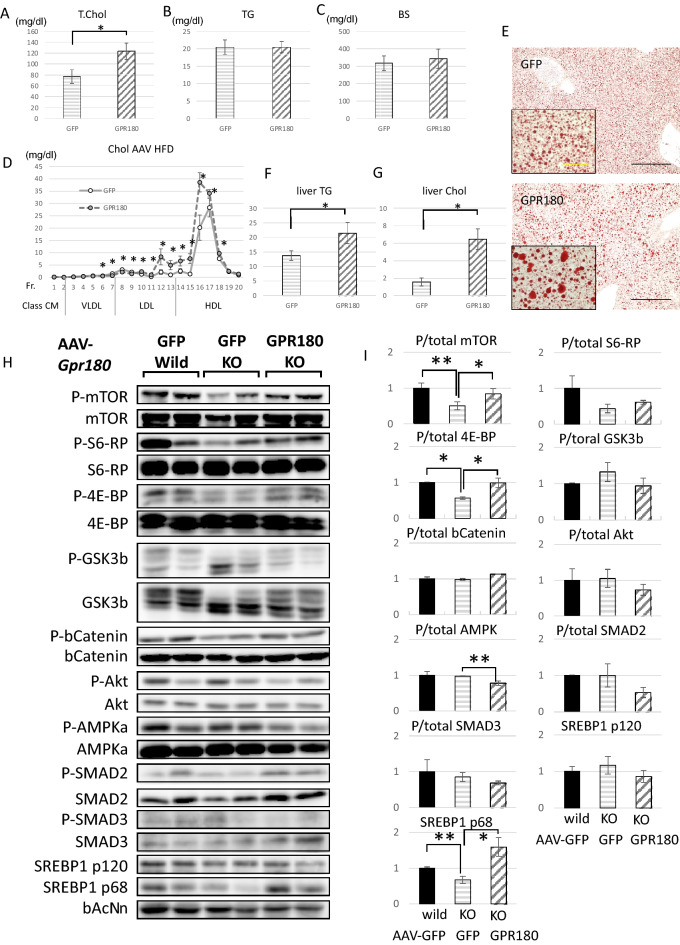
AAV-mediated hepatic rescue of GPR180 expression in KO mice. (**A**) Plasma total cholesterol, (**B**) TG and (**C**) blood glucose of AAV8-CAG-GFP or AAV8-CAG-GPR180 injected KO mice fed HFD after 6 h starvation (n = 6). (**D**) Cholesterol levels in HPLC-fractionated plasma of AAV8-CAG-GFP or AAV8-CAG-GPR180 injected KO mice fed HFD (n = 4). (**E**) Representative Oil Red O staining of 16-week-old mouse liver fed HFD. Black and yellow scale bars indicate 100 and 20 μm, respectively. (**F**) TG and (**G**) cholesterol contents in liver extract (mg/g) of AAV8-CAG-GFP or AAV8-CAG-GPR180 injected KO mice fed HFD (n = 6). (**H**) Twenty micrograms of protein per sample of AAV8-CAG-GFP injected wild-type liver, AAV8-CAG-GFP or AAV8-CAG-GPR180 injected KO mouse liver were stained with the primary antibodies indicated on the left and HRP-labeled secondary antibody. (**I**) Relative band intensities against the normalized mean value of wild-type mice are shown as mean values with standard deviation (n = 6). *p < 0.05. **p < 0.01.

To exclude the possibility of fat accumulation resulting from extrahepatic causes, the AAV8 vector was used to deliver human *GPR180* cDNA to the livers of *Gpr180*KO mice. The mice showed partial recovery of plasma and hepatic lipid levels compared to mice injected with the control vector AAV8-GFP (Fig. 5A–G). Furthermore, AAV8-mediated GPR180 expression rescued phosphorylation of mTOR and 4E-BP and activation of SREBP1 protein with increased lipogenic mRNA levels in the livers of *Gpr180*KO mice fed HFD (Fig. 5H–I, Supplementary Fig. 3). These results suggested that hepatic expression of *Gpr180* enhanced mTORC1 signaling and SREBP1 activation, which resulted in hepatic lipid accumulation and increased plasma lipid levels.

## Discussion

Intron 6 of *GPC6* showed a genetic association signal in our GWAS for nonobese NAFLD^7^. The Encode database and chromosome conformation capture assay suggested that this region functions as a regulatory element for adjacent genes, in which *GPR180* was nominated as the most likely locus involved functionally in hepatic lipid levels. Although our report was the first to show *GPR180* as a novel candidate locus for NAFLD, previous trans-ancestry GWAS showed *GPR180* as a novel locus associated with plasma lipid levels^8^. The lead SNP of the study, rs1341267, was mapped to the 3’ noncoding sequence of *GPR180*, which was 27 kb downstream from intron 6 of *GPC6* and was not included in the linkage equilibrium block of our association signal. However, the trans-ancestry GWAS strengthened the functional involvement of *GPR180* in lipid metabolism demonstrated by this study.

Very recently, GPR180 was shown to be a component in TGFβ signaling for thermogenic brown adipocytes^9^. Both the global and adipocyte specific *Gpr180*KO mice showed increased weight gain after HFD due to decreased energy expenditure, which was suggested to result from reduced expression of uncoupling protein (UCP) via reduction of TGFβ signaling. The decreased energy expenditure in *Gpr180*KO mice was accompanied by impaired glucose tolerance and hepatic lipid accumulation at a regular housing temperature (22 °C) but not at a nonthermogenic temperature (30 °C)^9^. In contrast to this study, our *Gpr180*KO mice did not show excessive weight gain and glucose intolerance; rather, hepatic lipid deposition was ameliorated under regular temperature. Furthermore, AAV8-mediated hepato-directional *Gpr180* KD also attenuated hepatic lipid, while and hepato-directional rescue in our global *Gpr180*KO mice recovered lipid accumulation. Although, it was difficult to estimate what caused these phenotypic discrepancies among the global *Gpr180*KO mice, the most distinctive difference was the target of guide RNA of CRISPR/Cas9, exon 3 vs. intron 5 and 6 of our study. Differences in the truncated protein, expression levels, off-target editing and unknown differences in the background mouse strain are suggested as potential causes for the phenotypic discrepancies among the *Gpr180*KO mice. However, in wild-type mice, GPR180-induced thermogenic energy expenditure in adipocytes is not contradictory to hepatic lipogenesis. Lipid supply via lipoprotein to adipocytes is a principal energy source for thermogenesis in brown adipocytes, which is an adaptive response to a cold environment. Our *Gpr180*KO mice showed not only ameliorated hepatic lipid accumulation, but also reduced plasma lipid levels, suggesting that GPR180 increases lipid secretion from the liver under HFD. GPR180 has been shown to be expressed ubiquitously. In vascular endothelial cells, depletion of GPR180 abolished intimal thickening after mechanical injury^10^, suggesting the involvement in the cell proliferation signals. These preceding studies and our study indicate the pleiotropic function of GPR180 protein, which should be analyzed further using cell type specific KO mice.

It has been reported that KD of *Gpr180* weakened TGFβ signaling in adipocytes^9^. Reciprocally, phosphorylation of SMAD3, the principal signal transducer of the TGFβ receptor, was up-regulated by over-expression of *Gpr180*^9^. However, in the mouse liver phosphorylation of SMAD2 and 3 was not affected by Gpr180 expression levels. Furthermore, the TGFβ signaling pathway was not a significantly enriched gene set expressed in liver of HFD fed wild-type or *Gpr180*KO mice. Instead, the mTOR signaling pathway was the most plausible pathway for the hepatic lipid accumulation in the results of GSEA analysis. Phosphorylation analysis also supported the weakened mTOR signaling in the *Gpr180*KO mouse liver. Involvement of the mTORC1 pathway in lipid metabolism has been repeatedly reported^11–14^. Defects of components of the mTORC1 pathway downregulate SREBP activation and lipogenesis. Our *Gpr180*KO mice showed reduced levels of the activated form of SREBP1 under HFD in accordance with dephosphorylation of mTOR. The reduction of the activated form SREBPs was accompanied by reduced expression of fatty acid and cholesterol synthesis genes, suggesting that ameliorated hepatic lipid storage in *Gpr180*KO mice resulted from attenuated mTOR signaling.

mTOR integrates diverse environmental stimuli such as nutritional status and growth factors in systemic organs. Furthermore, it forms two distinct complexes by using unique accessory partners, mTORC1 and mTORC2, which are involved in individually unique functions but simultaneously form a feedback loop in glucose and lipid metabolism^15^. Akt is a mediator in the crosstalk between mTORC1 and mTORC2^16,17^, and is also an early effector in PI3K pathway (e.g. under insulin signaling)^15^, but *Gpr180*KO and *GPR180*KD reduced mTOR phosphorylation without significant deactivation of Akt. Furthermore, insulin obscured the reduction of mTOR phosphorylation by *GPR180*KD. These results suggested that lack of GPR180 selectively ameliorated mTORC1 signaling. The global inhibitor of mTORC, rapamycin, is ineffective for ectopic lipid accumulation^15^. In this context, GPR180 could be a novel therapeutic target for fatty liver and dyslipidemia. In adipocytes, collagen triple helix repeat containing 1 (CTHRC1) was shown to be a physiological ligand for GPR180, which enhanced energy expenditure in mice^9^. Although, further studies are required to clarify whether CTHRC1 acts as an agonist for GPR180 in the liver, the development of antagonists is needed for the treatment of steatosis.

## Methods

### Chromosome conformation capture assay and database survey

The functional domain in the association signal in intron 6 of GPC6 was searched using the databases ENCODE^18^ (http://genome.ucsc.edu/ENCODE/) and HiC^19^ (http://3dgenome.fsm.northwestern.edu/view.php). To explore the functional partner of the DNA fragment in the GWAS association signal, a quantitative chromosome conformation capture assay (3C-assay) was carried out according to the published protocol^20^ with modification of the quantification procedure. Instead of qPCR, we quantified the intermolecularly ligated DNA fragments of Huh-7 and HEK293 cells using low-cycle PCR and subsequent pair-end sequencing with the Nextera and the MiSeq system (Illumina, San Diego, CA). The primers used are listed in Supplementary Table 1.

### Adeno-associated virus (AAV) vector constructs

The shRNA templates of mouse *Gpc6*, *Dct*, *Tgds* and *Gpr180* (Supplementary Table 1) were inserted in the pAAV-U6-CMV-hrGFP vector^21^. The open reading frame of human *GPR180* cDNA was amplified from cDNA of Huh-7 cells and inserted in the pAAV-CAG vector^22^. Recombinant AAV8 vectors were produced by calcium phosphate transfection of human embryonic kidney HEK-293 T cells using a helper-virus-free system, and purified twice with CsCl_2_ density gradients. AAV titer was determined by quantitative PCR. Male mice were injected *i.v.* with AAV at 1.00E + 11 VG/mouse.

### Mouse experiments

All mice were housed in a standard environment (22 °C, 12 h light/dark cycle, dark phase starting at 8:00 pm, 25% humidity), with ad libitum access to ND (23% protein, 2.8% fiber, 5.1% fat, 5.8% ash; Oriental Yeast Co., Ltd., Tokyo, Japan) and water. In MCDD cohorts (18.3% protein, 5.0% fiber, 23.5% fat; Oriental Yeast Co., Ltd.), the feeding regimen was initiated at 12 weeks of age and continued for one week. In HFD cohorts (29.5% protein, 4.9% fiber, 32% fat; CLEA Japan, Inc., Tokyo, Japan), the feeding regimen (8–12 weeks) was initiated at 8 weeks of age. Blood samples were collected from the tail vein after 4 h of starvation, and plasma TG and total cholesterol concentrations were measured using the Fuji Dry Chem system (Fujifilm, Kanagawa, Japan). TG and cholesterol in lipoprotein fractions were determined using the LipoSEARCH HPLC system (Skylight Biotech Inc., Akita, Japan). For the *i.p.* glucose tolerance test, mice were fasted for 6 h and fasting glucose levels were obtained from the tail vein using a glucometer (ACCU-CHEK; Roche diagnostics, Tokyo, Japan). d-glucose (Sigma-Aldrich) was *i.p.* injected at a dose of 1 mg/g body weight and blood glucose levels were measured 15, 30, 60, 90 and 120 min after glucose injection using the glucometer. Liver lipids were extracted using Folch’s method in chloroform/methanol from liver specimens after decapitation under anesthesia, and TG and cholesterol were measured using a LabAssay Triglyceride Kit (Wako, Osaka, Japan) and Cholesterol E-test Kit (Wako), respectively. Frozen sections of mouse livers were stained with Oil Red O, and the stained area was measured using microscope-associated software (KEYENCE Japan, Osaka, Japan). The Animal Care and Use Committee of Jichi Medical University approved all procedures involving animals. All experiments were performed in accordance with guidelines and regulations of Jichi Medical University and ARRIVE guidelines (https://arriveguidelines.org).

### Generation of Gpr180 global knockout

The *Gpr180* null allele was obtained by CRISPR/Cas9 based genome editing, which was carried out at the Laboratory Animal Resource Center, University of Tsukuba (https://www.md.tsukuba.ac.jp/LabAnimalResCNT/). A bicistronic expression vector (pX330; Addgene, Watertown, MA) was used to express Cas9 and an sgRNA, which targeted a sequence (CCA)TGCAGCTCGTCATTTGTCAC (PAM sequence in brackets) in intron 5 or TGAAATAATCGCCTTAATCC(GGG) in intron 6 of the *Gpr180* gene (Supplementary Fig. 1). Double strand break and direct ligation at these target sites was predicted to delete exon 6 of *Gpr180* mRNA (NM_021434), which simultaneously resulted in a frame shift. The truncated mRNA encodes N-terminal 245 amino acids of wild-type *Gpr180* mRNA (NP_067409, 441 residues), and an early termination codon appears at the following 10th codon. The constructs were simultaneously introduced to fertilized eggs of C57BL/6J mice (Charles River Laboratories, Yokohama, Japan) by electroporation. The founder mice were screened by genotyping PCR using DNA obtained by tail biopsy. To detect the deletion of exon 6, two primer sets were used: Gpr180int5Fw; TGTGCTTCTACGGCAGGTGA, and Gpr180int6Rv; GAATGACTTTTAGGAAGCAGT (flanking sequences of guide RNAs), and/or Gpr180ex6Fw; GACATTGCCTCCCAAATTCA and Gpr180ex6Rv; CTCACCTGTGTGATGACGAT (complementary sequences of exon 6).

### Expression profiling

Mouse hepatic total RNA was extracted immediately after euthanasia. A TruSeq mRNA sample preparation kit (Illumina) was used for the preparation of sequence libraries. Sequencing was performed as 75 bp, single reads and dual index reads on an Illumina NextSeq500 instrument. Approximately 15–25 million reads per sample were obtained. The output fastq row data were assembled to the mouse genome (mm10) using the TopHat Alignment application (Illumina). The output FPKM data were further analyzed using gene set enrichment analysis software (GSEA; Broad Institute)^23^ with a hallmark gene set collection^24^ as a gene set database. The nominated or suspected molecular signatures were studied by western blotting of mouse liver proteins using the following specific primary antibodies: p-mTOR (#5536; CST, Danvers, MA), mTOR (#2983; CST), p-GSK-3b (#5558; CST), GSK-3b (#12456; CST), p-βCatenin (#9561; CST), βCatenin (#9582; CST), p-Akt (#5012; CST), Akt (#58295; CST), p-AMPKα (#2535; CST), AMPKα (#5832; CST), p-SMAD2 (#3108; CST), SMAD2 (#5339; CST), p-SMAD3 (#9520; CST), SMAD3 (#9523; CST), p-S6 (#2215; CST), S6 (#2217; CST), p-4E-BP (#2855; CST), 4E-BP (#9644; CST), and SREBP-1 (ab28481; Abcam, Cambridge, MA). The molecular signatures were also studied in Huh-7 cells with or without *GPR180* knockdown (KD) using the pAAV-U6-CMV-hrGFP vector, which was validated by western blotting stained by anti-human GPR180 (R12-2843; Assay Biotechnology Company, Sunnyvale, CA). Huh-7 cells transfected with control or KD vector were cultured for 24 h and stimulated by insulin (100 nM) or vehicle for 30 min before the harvest. The band intensities of western blotting were quantified using ImageQuant LAS 4000 (GE Healthcare, Piscataway, NJ). Relative RNA expression levels in Huh-7 cells were assessed by RT-qPCR using the primers listed in Supplementary Table 1.

### Statistical analysis

The mouse studies used at least 6 animals as replicates and the data were evaluated using Student’s t-test. Statistical analysis was performed using R v3.6.0. For all analyses, statistical significance was taken at a P value of < 0.05.

## Supplementary Information

Supplementary Information.

## Data Availability

The datasets generated during the expression profiling are available in the NCBI’s Gene Expression Omnibus repository accession, GSE211832.

## Abbreviations

NAFLD: Nonalcoholic fatty liver disease
AAV: Adeno-associated virus
GWAS: Genome-wide association study
MCDD: Methionine and choline-deficient diet
HFD: High-fat diet
GPR180: G protein-coupled receptor 180
