# Supplementary material for: Lack of GPR180 ameliorates hepatic lipid depot via downregulation of mTORC1 signaling
Source: Sci Rep. 2023 Feb 1;13:1843. doi: 10.1038/s41598-023-29135-5 (PMC9892563; doi:10.1038/s41598-023-29135-5)
Supplement: Supplementary file 1 — Supplementary Information. [file 41598_2023_29135_MOESM1_ESM.pdf]

Supplementary material

Lack of GPR180 ameliorates hepatic lipid depot via downregulation of mTORC1 signaling

Ken Yoshida[1,2], Kazuha Yokota[1,3], Kazuhisa Watanabe[1], Ayumi Matsumoto[1], Hiroaki Mizukami[4], Sadahiko Iwamoto[1]

Supplementary Figure 1. Relative mRNA levels of target genes in mice livers administrated with AAV-knockdown vectors. Relative mRNA levels in mice liver against the normalized mean value of mice injected with negative control vector (Contrrol) are shown as mean values with standard deviation (n=4). * indicates p < 0.05. ** indicates p < 0.01.

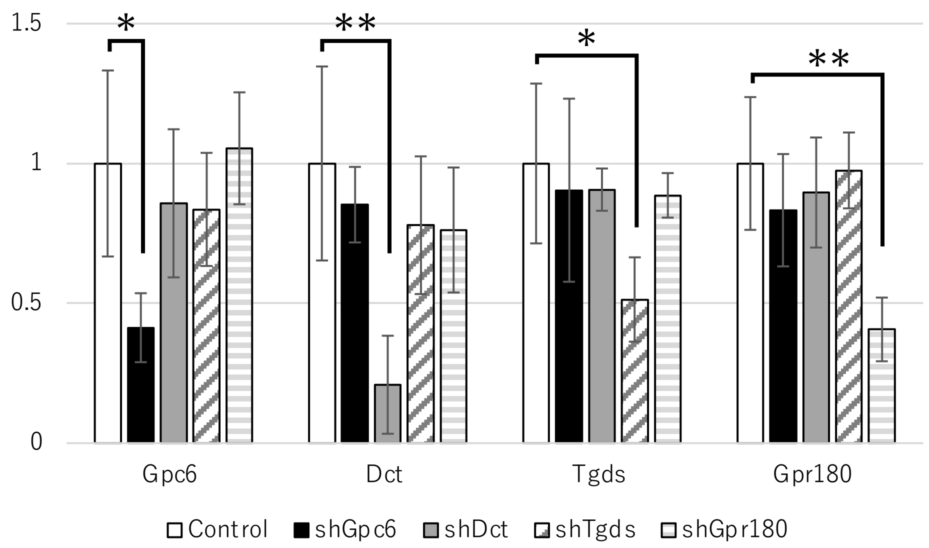

Supplemantary Figure2. Strategy for the generation of global Gpr180 knockout mice. A. Genomic organization of mouse Gpr180 and position of the guide RNA of CRISPR/Cas9 vector. B. Left and right target sequences of CRISPR/Cas9 with targets number estimated by CRISPRdirect (https://crispr.dbcls.jp). C. Representative PCR screening of founder mice using primers of Gpr180int5Fw and Gpr180int6Rv. KO allele bands encoded the double strand break and the direct ligation at left and right target sites. D. Relative mRNA levels in wild and KO mice organs normalized against housekeeping gene, 36b4. Muscle; gastrocnemius muscle, PK fat; peri-kidney fat pad, SC fat; inguinal subcutaneous fat pat. E. Representative RNA sequence data around *Gpr180* visualized by Integrative Genomics Viewer v2.8.2.

A

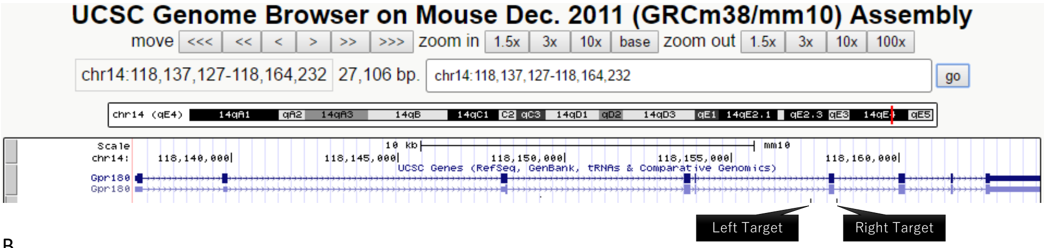

B

Left target sequence	number of target sites		
20mer+PAM (total 23mer)	20mer +PAM	12mer +PAM	8mer +PAM
CCATGCAGCTCGTCATTTGTCAC	1	6	6913
Right target sequence	number of target sites		
20mer+PAM (total 23mer)	20mer +PAM	12mer +PAM	8mer +PAM
TGAAATAATCGCCTTAATCCGGG	1	2	2263

C

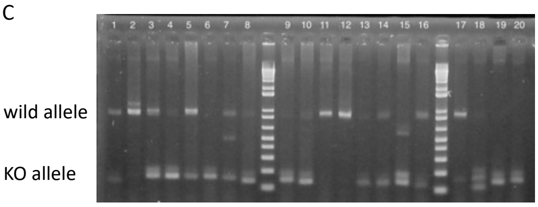

D

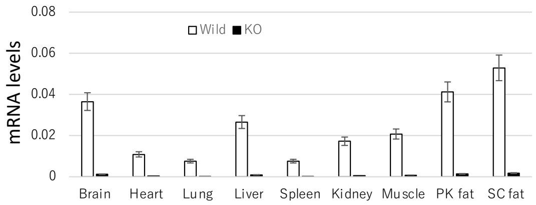

E

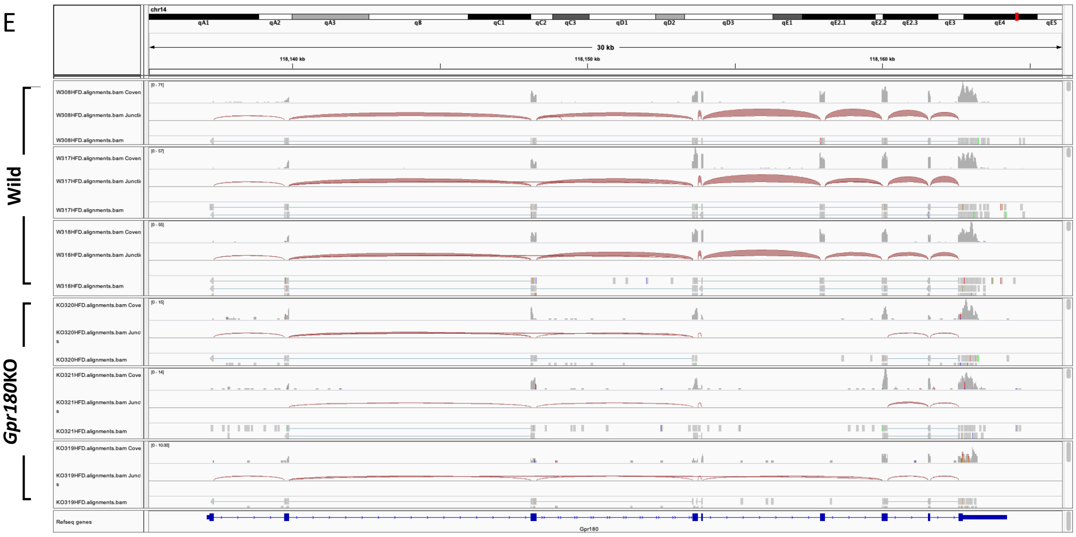

Supplementary Figure 3. Relative mRNA levels of lipid metabolisms in mice liver. Relative mRNA levels in mice liver against the normalized mean value of wild mice fed ND (WT ND) are shown as mean values with standard deviation (n=6). * indicates $p < 0.05$. ** indicates $p < 0.01$.

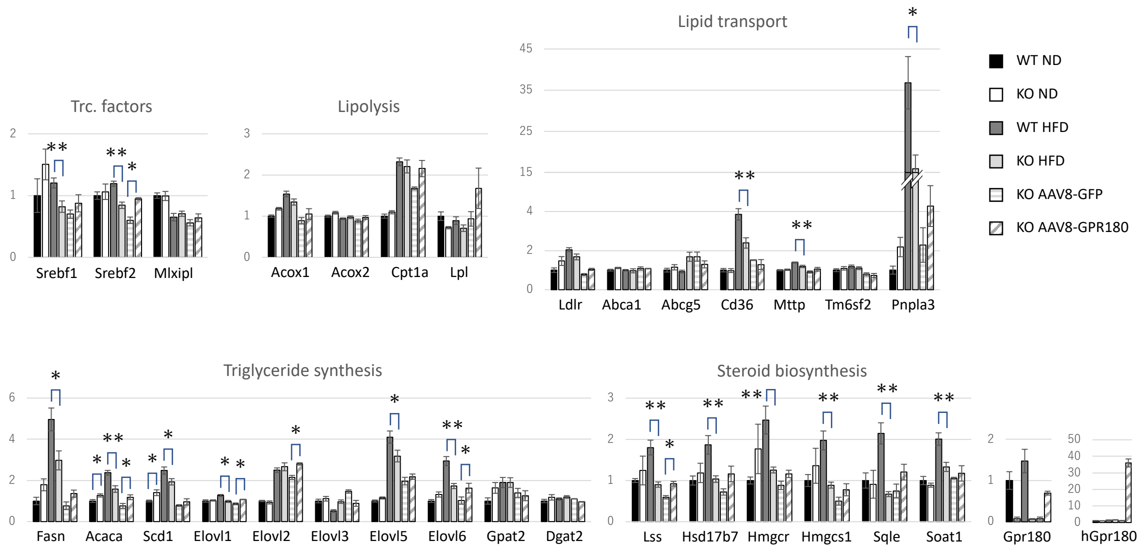

supplementary table. Oligonucleotide sequences used in this study

**Knockdown template**	**sequence**
shGpc6Top	GATCCGAGCTAAAGCGGTACTACATTCAAGAGATGTAGTACCGCTTTAGCTCTTTTTTGGAAA
shGpc6Bot	AGCTTTTCCAAAAAAGAGCTAAAGCGGTACTACATCTCTTGAATGTAGTACCGCTTTAGCTCG
shDctTop	GATCCGCAAGACAAGATGACCCAATTCAAGAGATTGGGTCATCTTGTCTTGCTTTTTTGGAAA
shDctBot	AGCTTTTCCAAAAAAGCAAGACAAGATGACCCAATCTCTTGAATTGGGTCATCTTGTCTTGCG
shTgdsTop	GATCCGTCTTACTGGGAAAGGTACTTCAAGAGAGTACCTTTCCCAGTAAGACTTTTTTGGAAA
shTgdsBot	AGCTTTTCCAAAAAAGTCTTACTGGGAAAGGTACTCTCTTGAAGTACCTTTCCCAGTAAGACG
shGpr180Top	GATCCGGATCATTTTAGCGCCAGATTCAAGAGATCTGGCGCTAAAATGATCCTTTTTTGGAAA
shGpr180Bot	AGCTTTTCCAAAAAAGGATCATTTTAGCGCCAGATCTCTTGAATCTGGCGCTAAAATGATCCG
HumShGPR180Top	GATCCGGAAAAGAAGCTAAACTCTACTTCAAGAGAGTAGAGTTTAGCTTCTTTTCCTTTTTTGGAAA
HumShGPR180Bot	AGCTTTTCCAAAAAAGGAAAAGAAGCTAAACTCTACTCTCTTGAAGTAGAGTTTAGCTTCTTTTCCG

**primers for 3C assay**	**sequence**			**sequence**
T1	FwAdaptor-CCATGCAGAAATCACTGGGT		GPC6	RvAdaptor-TCTTGGGGATGTGTGTGCGT
T2	FwAdaptor-CACTGTGTATCTGTCTGGGT		GPC6-AS2	RvAdaptor-GAGGTTATCAAATGCCTCTG
T3	FwAdaptor-ATCTCTGAGTTGTCCACCTA		DCT	RvAdaptor-TATCGTGGACGGAGAGTCTT
T4	FwAdaptor-ACCTCCCAAGAGGCAGAAAC		TGDS	RvAdaptor-CGGATGGTAGGGGTATGGAA
T5	FwAdaptor-CTCAAGAGAGCTGAACAGCT		GPR180	RvAdaptor-TTAAGACAGGTATAGCCAGCA
T6	FwAdaptor-CCCGATAGGTCCTTTTACTC			
T7	FwAdaptor-GTGTCCTCGAGCACTCTAGT			
(FwAdaptor)	TCGTCGGCAGCGTCAGATGTGTATAAGAGACAG			
(RvAdaptor)	GTCTCGTGGGCTCGGAGATGTGTATAAGAGACAG			

**qPCR primers**	**sequence**		**qPCR primers**	**sequence**
GPR180fw	CGCTTCCCAAATTCAGATGT		GPR180rv	CCACTGGAGAGGTCTGCTTT
SREBF1fw	GCCATTGAGAAGCGCTACCG		SREBF1rv	CCTTGCGCAAGACAGCAGAT
SREBP2fw	AAGTCTGGCGTTCTGAGGAA		SREBP2rv	TTCAGCACCATGTTCTCCTG
MLXIPLfw	TGGGTGTTCAGCATCCTCATC		MLXIPLrv	CAGCCAGGCCAGTGAGGTCT
ACOX1fw	TTCTCAACAGCCCAACTGTG		ACOX1rv	GCCAGGACTATCGCATGATT
ACOX2fw	CCACCCAGGAGTTTGTGATA		ACOX2rv	CCCAAGTCTCCAGGCCACCA
CPT1Cfw	CCTGTGCAGCAGACACACCT		CPT1Crv	CATACCCATGGTCATGGGCA
LPLfw	ATTGGAATCCAGAAACCAGT		LPLrv	AGTCCTCTCTCTGCAATCAC

LDLRfw	TGCAAGGCTGTGGGCTCCAT		LDLRrv	GGTGTACTCGCTCCGGTCCA
ABCA1fw	CCAGACAGTTGTGGATGTGG		ABCA1rv	CCTGTGTGAACGGGATTCTT
ABCG5fw	CAGCGACCAGGAGAGTCAGGA		ABCG5rv	ACGCTGAAGGGGAGGACGTG
CD36fw	GTGCTCTCCCTTGATTCTGC		CD36rv	GTGCTCTCCCTTGATTCTGC
MTTPfw	CAGGGTGGTCTAGCTATTGAT		MTTPrv	TTTCACTCGGGTTTTAGACTC
TM6SF2fw	ATGGAGTTCTACACCAAGGA		TM6SF2rv	CCATCCCAGTAGCAGATGAA
PNPLA3fw	CCAAAGACGAAGTCGTGGAT		PNPLA3rv	GTACGTTGTCACTCACTCCT
FASNfw	AGCCTGGCTGCCTACTACAT		FASNrv	GCCTGCAGCTGGGAGCACAT
ACACAfw	AGCACAGCTCCAGATTGCCA		ACACArv	GGAGATACCCCATACATCATAC
SCD1fw	GGCTTCCACAACTACCACCA		SCD1rv	GTGGTGAAGTTGATGTGCCAG
ELOVL1fw	CCAGTTTGTCCTGGTCTCAC		ELOVL1rv	GCCATACATCCAGATGAGGT
ELOVL2fw	TTGAACTGGATACCTTGTGG		ELOVL2rv	CACCAGCTGAGCCTGTGTGA
ELOVL3fw	CCTTCATCATCCTGCGTAAG		ELOVL3rv	AGCACTGTGCTGTGGTGGTA
ELOVL5fw	TCCAGATTGGATACATGATT		ELOVL5rv	TCCTTCAGGTGGTCTTTCCT
ELOVL6fw	GGTTTCCGAGTCTCCCGGAA		ELOVL6rv	GCCCATCAGCATCTGAGTGA
GPAT2fw	GATGCCTGTGCTTCGCGAGT		GPAT2rv	GCCACTCCCATTCTTGGAGA
DGAT2fw	GATGCCTGTGCTTCGCGAGT		DGAT2rv	GCCACTCCCATTCTTGGAGA
LSSfw	CTGGACTGCGGCTGGATCGT		LSSrv	AGGAGCAGCACAGCCTTCAA
HSD17B7fw	GTCAGCAACCTGCAGTCGGT		HSD17B7rv	ATATTTAGTTGTGGATTAGGCA
HMGCRfw	TGGGACCAACCTACTACCTC		HMGCRrv	TTATCTTTGCATGCTCCTTG
HMGCS1fw	AACATGAAGCTCAGAGAGGA		HMGCS1rv	ACCACGTTCCTTCAAAGAGT
SQLEfw	CTTACTGGTGGAGGAATGAC		SQLErv	GCATCATCATAAAGGTCAGG
SOAT1fw	GAGAGCACCTCCAGAACAAG		SOAT1rv	TTCTGATGTGGTCCACTTCA
AKT1fw	CACTCGGAGAAGAACGTGGT		AKT1rv	CCCGTCCTTGTCCAGCATGA
mTORfw	CCATAAGAAAACGGGGACCA		mTORrv	AGACGGTTTGGTGAAACCAG

**Mouse qPCR prim**	**sequence**			**sequence**
musGPR180fw	AGATCCCCTTTGAAATGGTG		musGPR180rv	GACGAGGAGGAAAAAGAACT
musDctFw	AACAACCCTTCCACAGATGC		musDctRv	TAGTCACCGGTGGGAAGAAG
musTgdsFw	GGAGAGCTGGGTGGACTATG		musTgdsRv	TGGGCTTCCATCCTAAACTG
musGpc6Fw	CTCCTGGAGAGGATGTTCCA		musGpc6Rv	AATGGCTTCAGCTGGTCTGT

Supplementary information of Fig.4A. Full-length blots. Left numerals indicate the size standards. Arrowheads indicate the stained bands by each antibody with expected molecular size, which were cropped and aligned in Fig.4A. The blot filters for mTOR, GSK3b, bCatenin and Akt were cut into strips before the staining with antibodies. AMPKa staining were performed by re-probing after removal procedure of GSK3b antibodies, while the removal was incomplete.

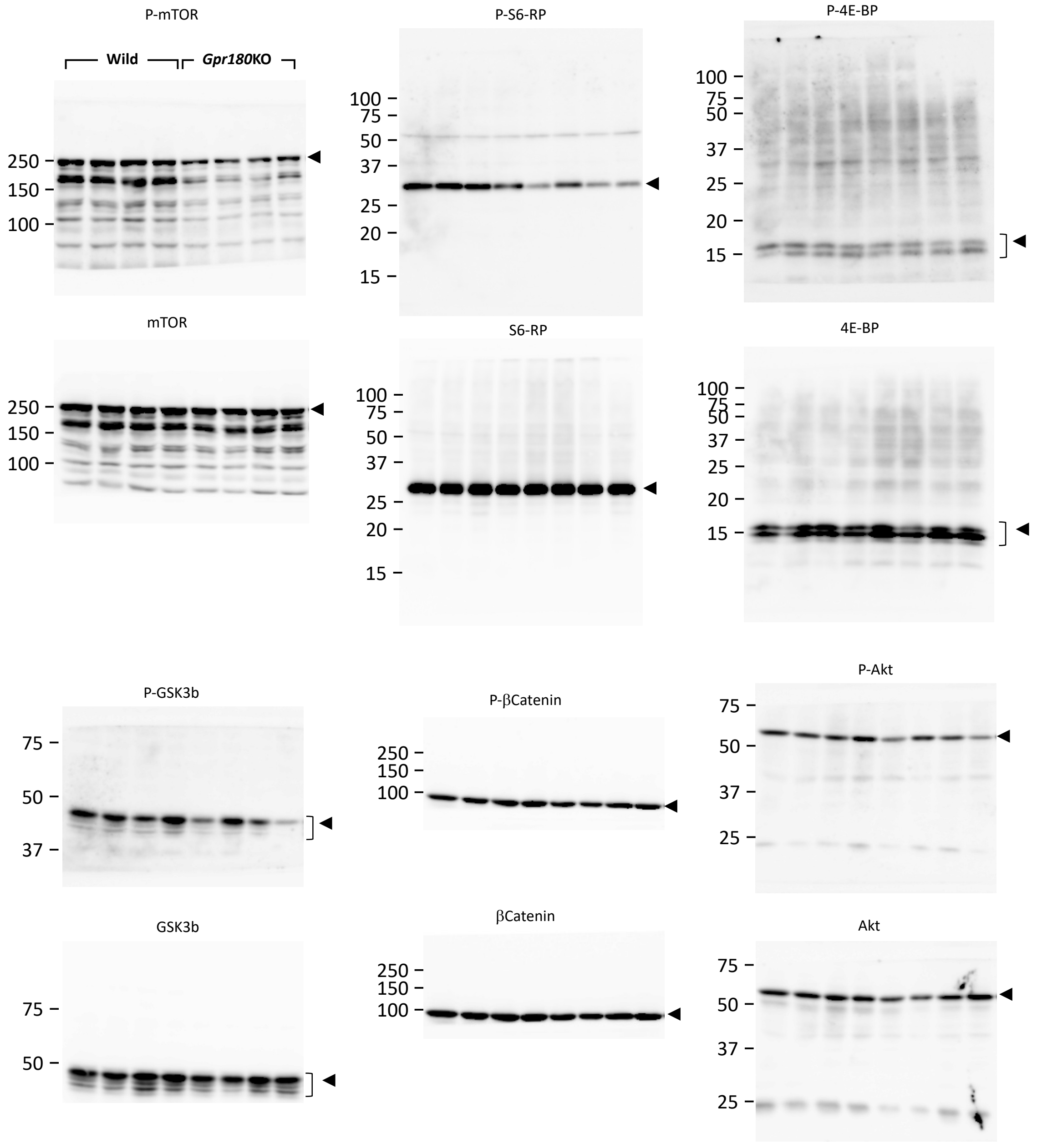

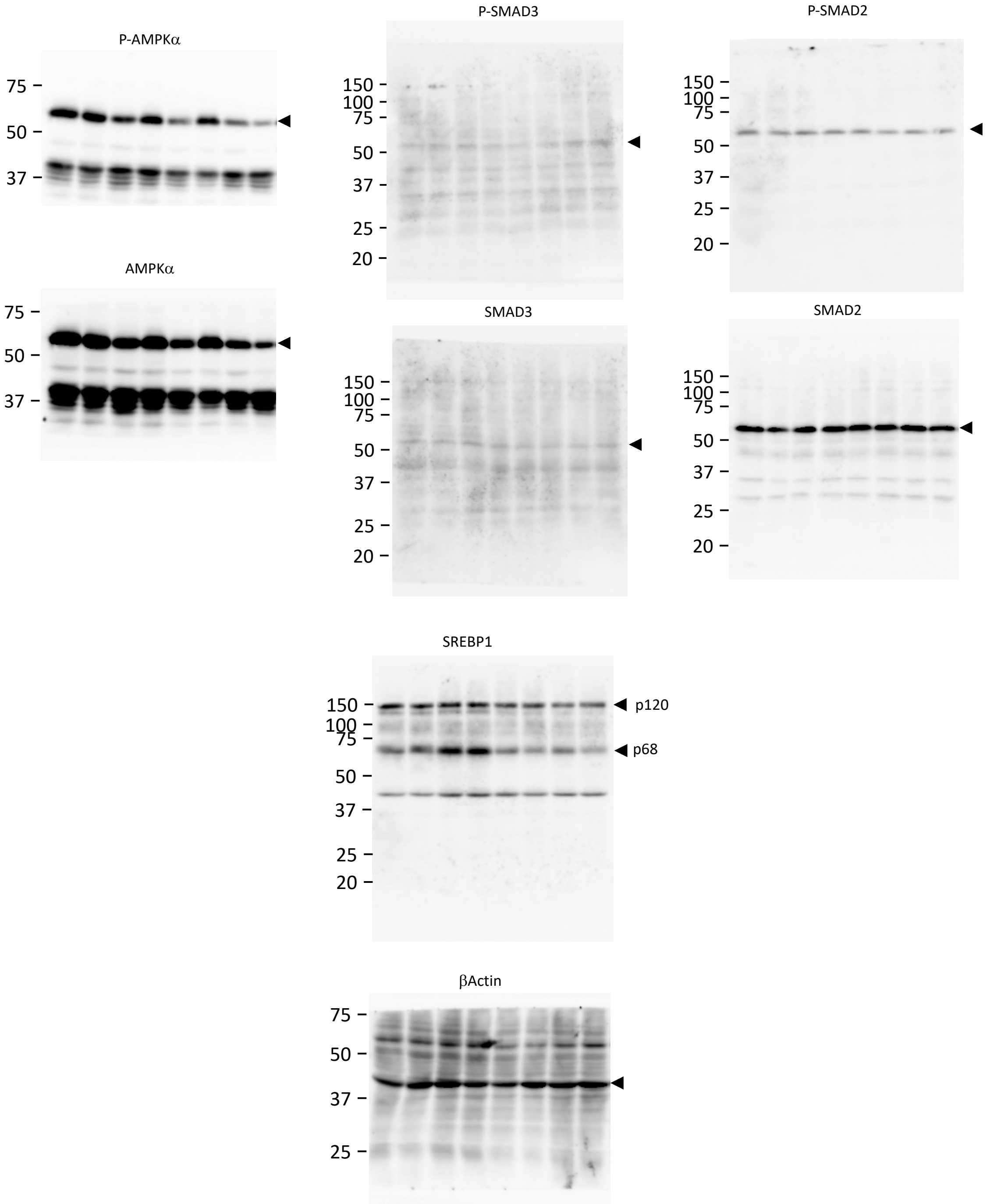
P-AMPKα
75
50
37
AMPKα
75
50
37
P-SMAD3
150
100
75
50
37
25
20
SMAD3
150
100
75
50
37
25
20
P-SMAD2
150
100
75
50
37
25
20
SMAD2
150
100
75
50
37
25
20
SREBP1
150
100
75
50
37
25
20
p120
p68
βActin
75
50
37
25

Supplementary information of Fig.4C. Full-length blots. Left numerals indicate the size standards. Arrowheads indicate the stained bands by each antibody with expected molecular size, which were cropped and aligned in Fig.4C. The blot filters for mTOR and Akt were cut into strips before the staining with antibodies.

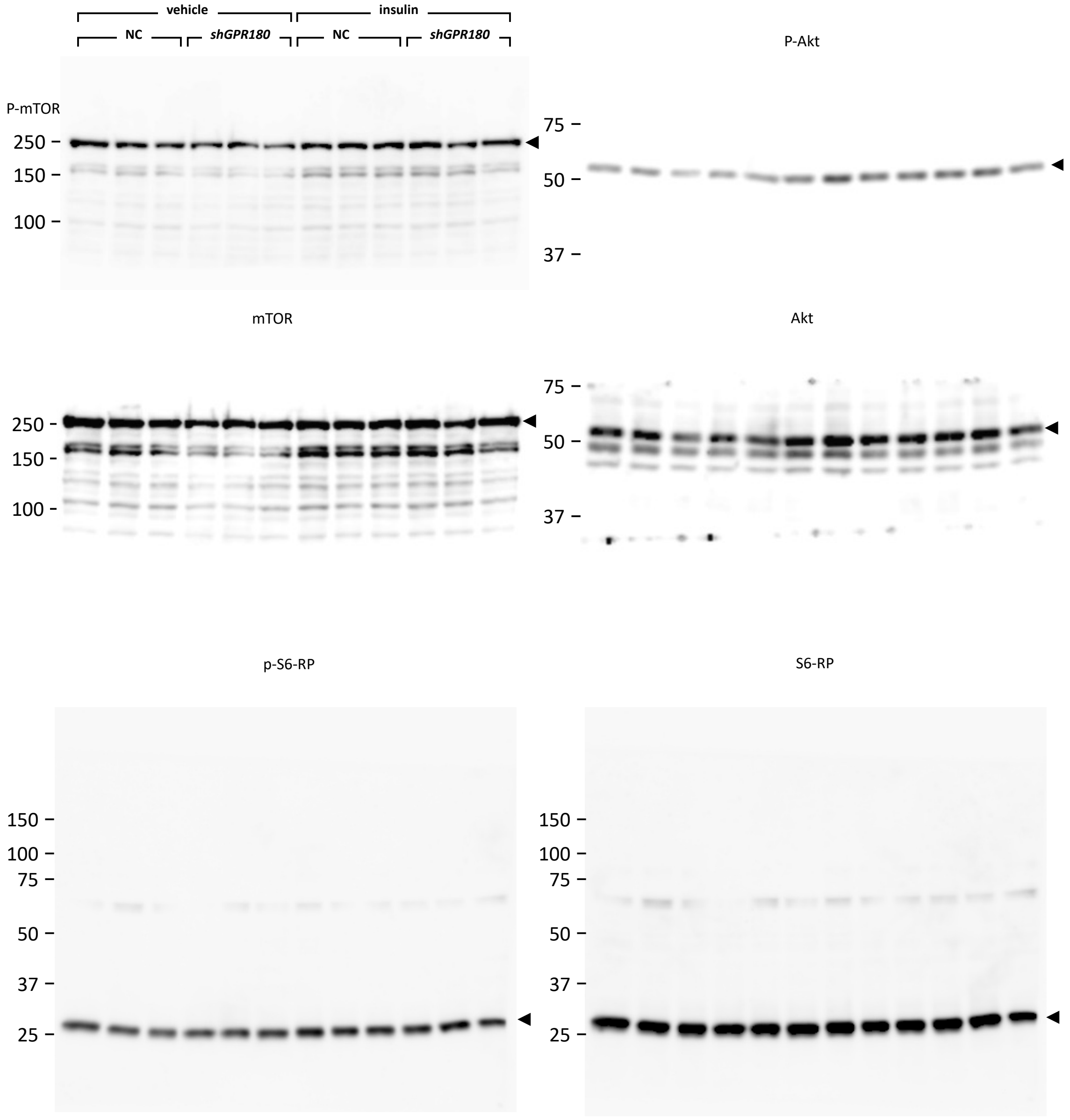

GPR180

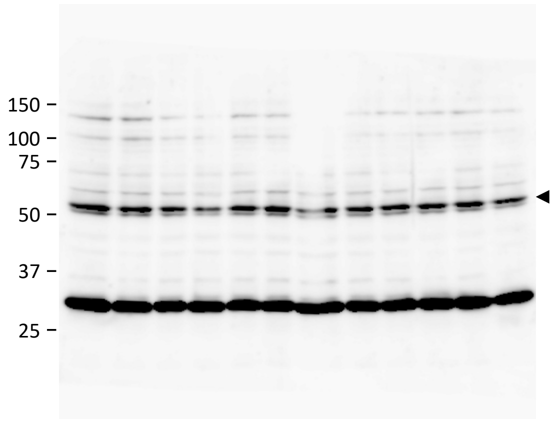

βActin

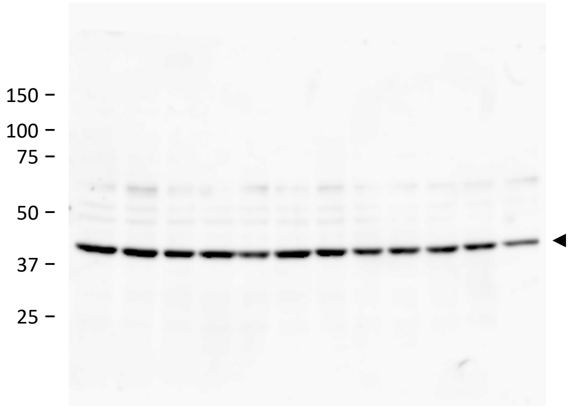

Supplementary information of Fig.5H. Full-length blots. Left numerals indicate the size standards. Arrowheads indicate the stained bands by each antibody with expected molecular size, which were cropped and aligned in Fig.5H. The blot filters for mTOR, GSK3b, bCatenin and Akt were cut into strips before the staining with antibodies.

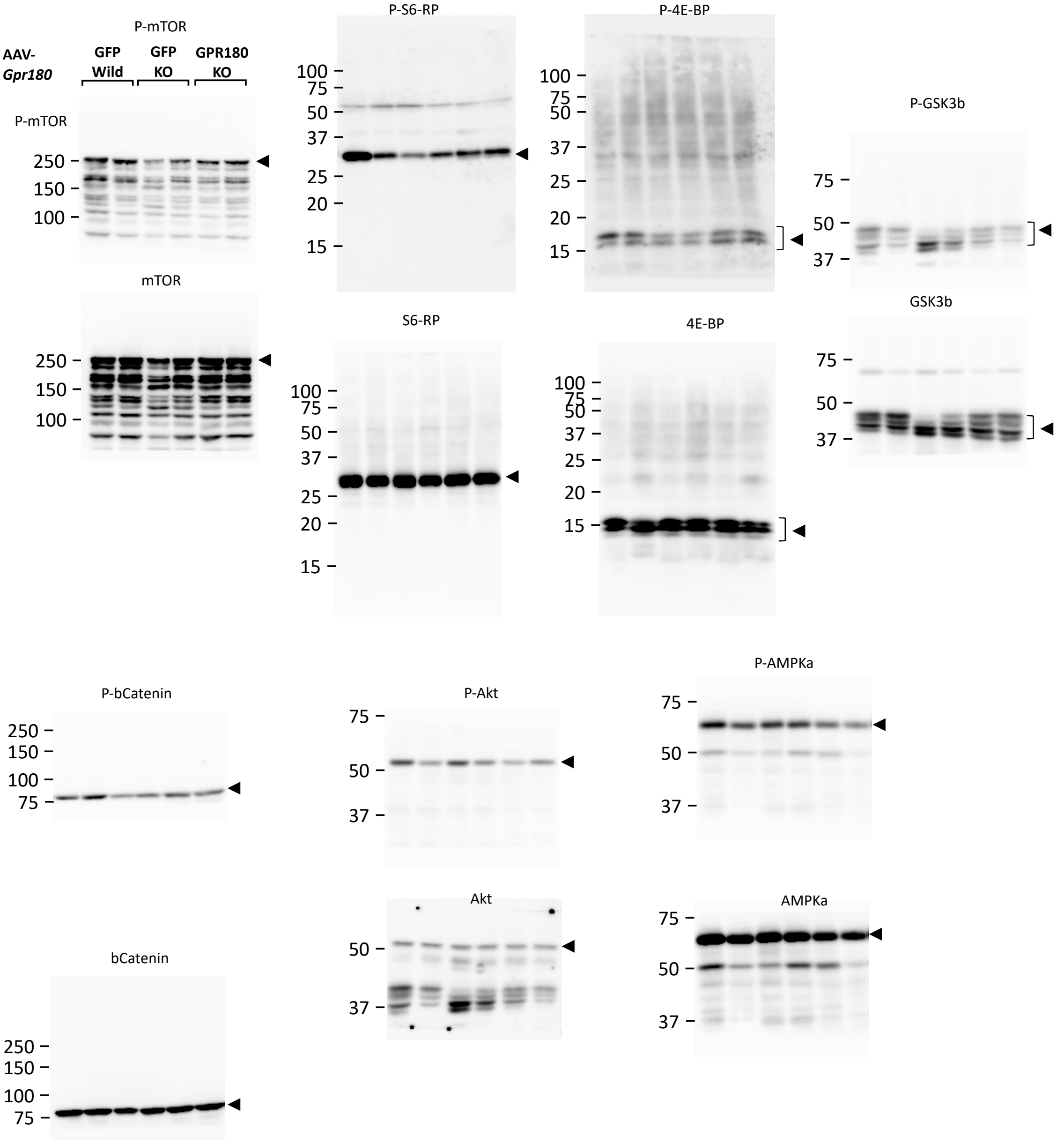

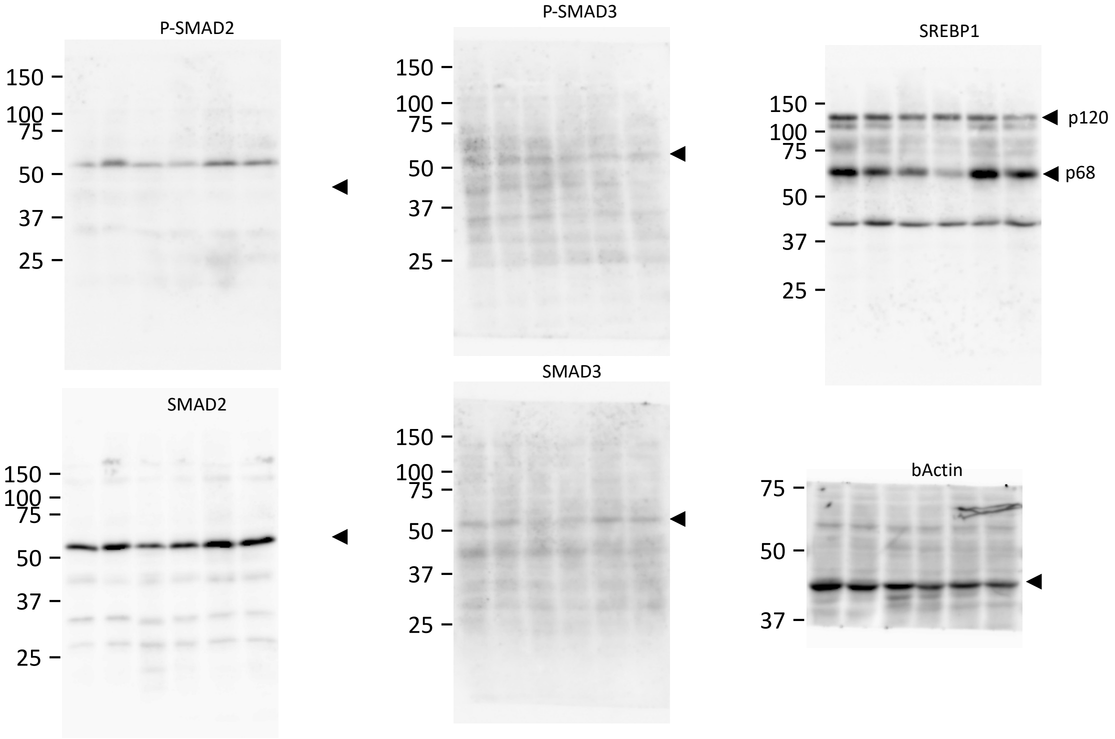
P-SMAD2
150
100
75
50
37
25
P-SMAD3
150
100
75
50
37
25
SREBP1
150
100
75
50
37
25
p120
p68
SMAD2
150
100
75
50
37
25
SMAD3
150
100
75
50
37
25
bActin
75
50
37
